# Supplementary material for: Rebuilding the autoimmune-damaged corneal stroma through topical lubrication
Source: bioRxiv. 2024 Dec 3:2024.11.29.626078. Preprint. [Version 1] doi: 10.1101/2024.11.29.626078 (PMC11642755; doi:10.1101/2024.11.29.626078)
Supplement: 1 [file NIHPP2024.11.29.626078V1-supplement-1.pdf]

bioRxiv preprint doi: https://doi.org/10.1101/2024.11.29.626078; this version posted December 3, 2024. The copyright holder for this preprint (which was not certified by peer review) is the author/funder, who has granted bioRxiv a license to display the preprint in perpetuity. It is made available under aCC-BY 4.0 International license.

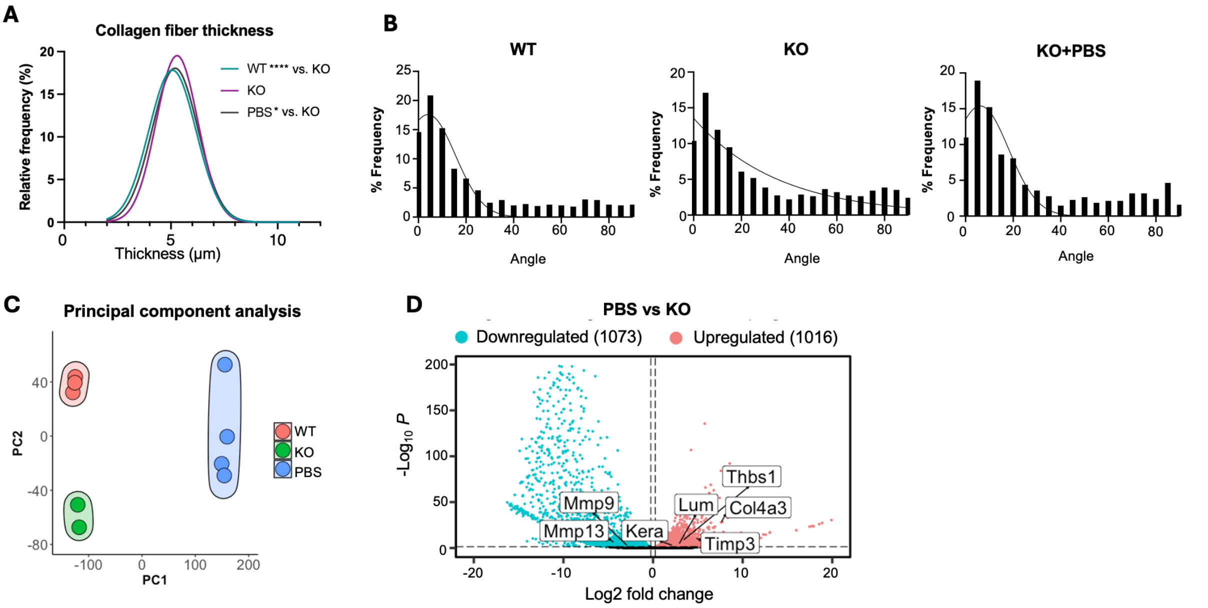

Supplementary Figure S1. Topical lubrication of the ocular surface restores the basement membrane and promotes stromal regeneration, related to Figure 1. A-B. Distribution of the collagen 1 fiber thickness (**A**) and alignment angle in relation to the BM (**B**) in the corneal stroma of each group. **C.** Principal component analysis (PCA) of the different treatment groups. **D.** Volcano plot of differentially expressed corneal genes in response to PBS versus untreated KO after 24hr of treatment. Red and blue dots represent significantly upregulated and downregulated genes, respectively (log2 FC ≥1 and padj <0.05). Data in B were subjected to a Kolmogotrov-Smirnov test. Each dot in the bar graph represents a biological replicate. n > 4 mice per group except for 24hr RNAseq analysis where n ≥ 2.

bioRxiv preprint doi: https://doi.org/10.1101/2024.11.29.626078; this version posted December 3, 2024. The copyright holder for this preprint (which was not certified by peer review) is the author/funder, who has granted bioRxiv a license to display the preprint in perpetuity. It is made available under aCC-BY 4.0 International license.

% cells in population

Cluster	WT	KO	PBS
0	0.00	61.31	0.53
1	0.00	38.61	0.31
2	61.56	0.00	0.59
3	0.00	0.00	35.83
4	28.26	0.00	0.48
5	0.03	0.00	25.24
6	0.00	0.00	18.02
7	0.00	0.00	15.21
8	10.15	0.00	0.17
9	0.00	0.08	3.63

Supplementary Figure S2. Table summarizing the proportion of keratocytes in each cluster within the different groups, related to Figure 2.

bioRxiv preprint doi: https://doi.org/10.1101/2024.11.29.626078; this version posted December 3, 2024. The copyright holder for this preprint (which was not certified by peer review) is the author/funder, who has granted bioRxiv a license to display the preprint in perpetuity. It is made available under aCC-BY 4.0 International license.

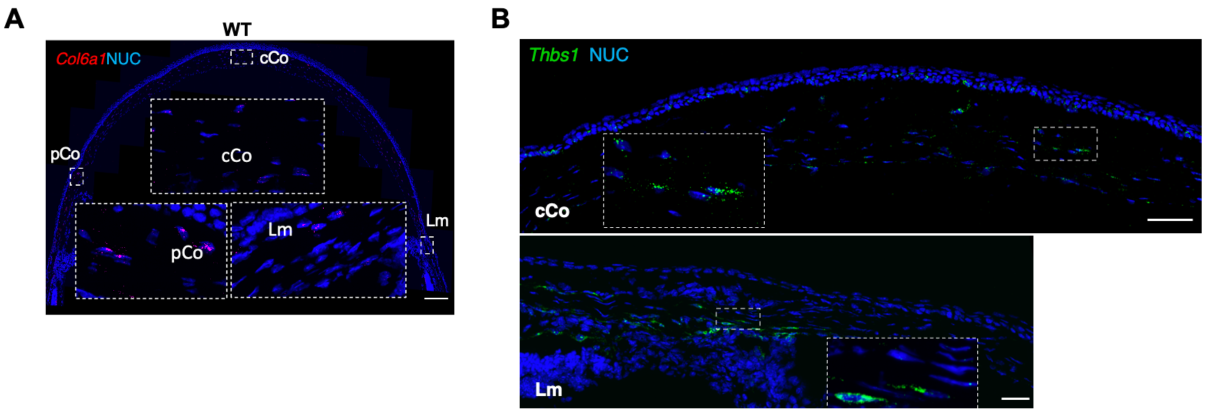

Supplementary Figure S3. Spatial locations of *Col6a1+* and *Thbs1+* cells in the central, peripheral and limbal regions of the wild type (WT) cornea, related to Figure 3. Scale bars = 50 µm.

bioRxiv preprint doi: https://doi.org/10.1101/2024.11.29.626078; this version posted December 3, 2024. The copyright holder for this preprint (which was not certified by peer review) is the author/funder, who has granted bioRxiv a license to display the preprint in perpetuity. It is made available under aCC-BY 4.0 International license.

A

WT Cluster	% cell population
C0	50.23
C1	23.57
C2	16.64
C3	9.56

Aire KO Cluster	% cell population
C0	30.45
C1	23.85
C2	8.32
C3	7.85
C4	7.29
C5	6.98
C6	6.65
C7	5.63
C8	2.86

PBS Cluster	% cell population
C0	36.93
C1	22.41
C2	20.48
C3	15.26
C4	4.91

B

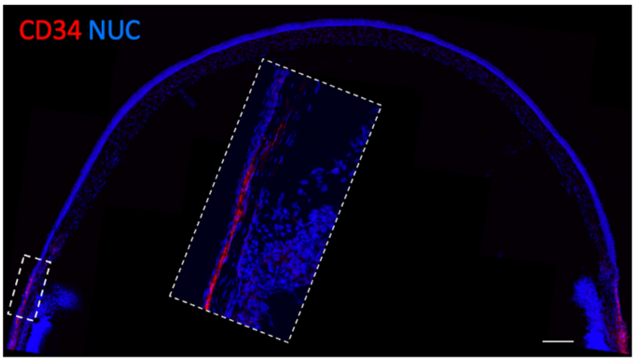

Supplementary Figure S4. Table summarizing the proportion of keratocytes in each cluster within the different groups identified by snRNAseq (**A**) and the location of CD34+ cells at the limbal region (**B**), related to Figure 4.

bioRxiv preprint doi: https://doi.org/10.1101/2024.11.29.626078; this version posted December 3, 2024. The copyright holder for this preprint (which was not certified by peer review) is the author/funder, who has granted bioRxiv a license to display the preprint in perpetuity. It is made available under aCC-BY 4.0 International license.

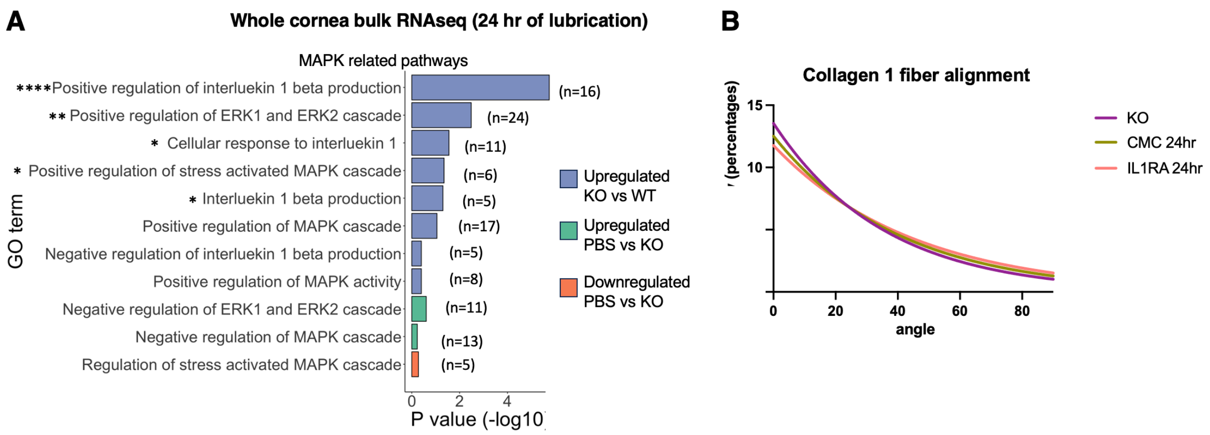

Supplementary Figure S5. Lubrication rescues stromal architecture via inhibition of IL1B-IL1R1-MAPK signaling, related to Figure 6. A. Gene ontology (GO) analysis highlighting alterations in IL1 and MAPK related pathways in WT, KO and KO+PBS stromal samples at 24 hr of treatment (bulk RNAseq). **B.** Frequency of collagen 1 fiber alignment, comparing KO, KO+CMC and KO+IL1RA at 24hr.
